# From Isolation to Inclusion: Circus Performances as Social Catalysts for Children with Medical Complexities

**DOI:** 10.31662/jmaj.2025-0276

**Published:** 2026-02-27

**Authors:** Emiko Karakawa, Yudai Kaneda, Akihiko Ozaki, Makoto Kosaka, Hiroyuki Beniya

**Affiliations:** 1 Department of Medical & Social Services, Orange Medical & Social Services Med.Co, Fukui/Nagano, Japan; 2 Clinical Research Center, Jyoban Hospital of Tokiwa Foundation, Fukushima, Japan; 3 Breast and Thyroid Center, Jyoban Hospital of Tokiwa Foundation, Fukushima, Japan; 4 Equally contributed to the work.

**Keywords:** children with medical complexity (CMC), social participation, inclusive design, international classification of functioning, Japan

## Abstract

Recent advances in medical care have increased the number of children with medical complexity (CMC) requiring daily support, yet their opportunities for social participation remain limited. We involved a 6-year-old CMC in circus performances held in three Japanese cities. Despite medical challenges, the child safely participated and experienced psychological growth through nervousness, ambition, and communication. This initiative required close interdisciplinary collaboration and highlighted the importance of trust between families and healthcare providers. Our experience illustrates how creative, community-based efforts can reduce barriers and promote inclusion for CMCs, contributing to a more equitable and supportive society.

Recent advances in medical technology have increased the number of children with medical complexity (CMC) needing daily medical support. In Japan, the number of CMCs using medical devices rose to 19,712 in 2018, doubling the 2008 figure ^(1)^. While the International Classification of Functioning, Disability and Health promotes the active societal participation of children with disabilities without waiting for functional improvements ^(2)^, CMCs tend to have far more limited opportunities for social interaction compared to their peers.

This disparity between the ideal of active societal participation and the lived experiences of CMCs underscores the practical obstacles they face, including the constant need for additional caregivers, dependence on medical devices, and a lack of accessible transportation―all of which limit their ability to engage in everyday social activities ^(3), (4)^. Despite these challenges, developing methods to support the social participation of CMCs not only directly benefits the children themselves but also strengthens trust between families and healthcare professionals, enhances family well-being, and ultimately fosters innovation in infrastructure, healthcare delivery, and inclusive design ^(3), (5)^. Proactive efforts in this area are therefore essential; however, the current knowledge and practical experience remain insufficient.

Since 2022, we have collaborated with circus artist Keisuke Kanai, who performed and choreographed at the Tokyo 2020 Paralympic Games Opening and Closing Ceremonies, to conduct artist-in-residence programs and workshops for CMC. From 2023, our team began participating in his stage production “Moonnight Circus,” which embodies the philosophy of “social circus,” where people of various backgrounds work together. Utilizing both the Paralympic Games legacy and medical expertise, we have produced inclusive performances featuring artists with disabilities, including CMC. This project has been supported by the Agency for Cultural Affairs and the Arts Council of Nagano, attracting over 3,500 attendees since its 2021 premiere.

Within this context, we undertook an initiative to involve a 6-year-old child with CMC in an aerial performance. The child had spinal muscular atrophy type I, chronic respiratory failure, gastrostomy, and right upper lobe atelectasis. He had severe motor impairment, requiring a buggy for mobility and full assistance in daily activities, while his cognitive development was appropriate for his age, though he was nonverbal. His daily medical care included mechanical cough assist, nighttime non-invasive positive-pressure ventilation, frequent sputum suctioning, and gastrostomy feeding. A total of six stages were held across three cities in 2024 ― Matsumoto (three times), Ueda (once), and Fukui (twice) ― with an approximate total audience of around 2,000 people, including caregivers and medical staff. Given the child’s medical condition, participation in these events required careful logistical coordination and substantial preparation.

To contextualize the logistical and medical burden required for participation, preparation for the approximately 90-minute performance began in April 2024 and extended over roughly nine months. A total of seven practice sessions were conducted, each lasting three to four hours, along with five venue-based run-through rehearsals held in the days preceding the performances. However, due to the long travel distance and occasional episodes of fever, the child was unable to attend some of these run-throughs. Participation required repeated long-distance travel, amounting to approximately 420 km in total; single-trip travel times ranged from 50 minutes to more than 2.5 hours, depending on the venue. During travel, his mother and our staff alternated in assisting him with meals and toileting, providing uninterrupted support throughout the journey. The family also stayed overnight for three nights during the Matsumoto shows and another three nights during the Fukui engagement. Rehearsal sessions generally lasted two to four hours, whereas full performance days required six to 10 hours on site, including medical preparation, aerial practice, and post-performance care.

The performances were based on the concept of “Nouveau Cirque (New Circus),” integrating traditional acrobatics such as aerial arts, pole dance, pantomime, and juggling with elements of theater, dance, music, and street culture. Performers included professional artists with disabilities, such as wheelchair users and deaf artists, as well as community members of various ages and abilities selected through open auditions since 2023. Figure 1 shows the child performing on aerial tissue, suspended approximately five meters above the ground, moving his legs like a fish tail to create rotation. The gesture of pointing upward with his fingers toward the ceiling was his own original choreography.

**Figure 1.**
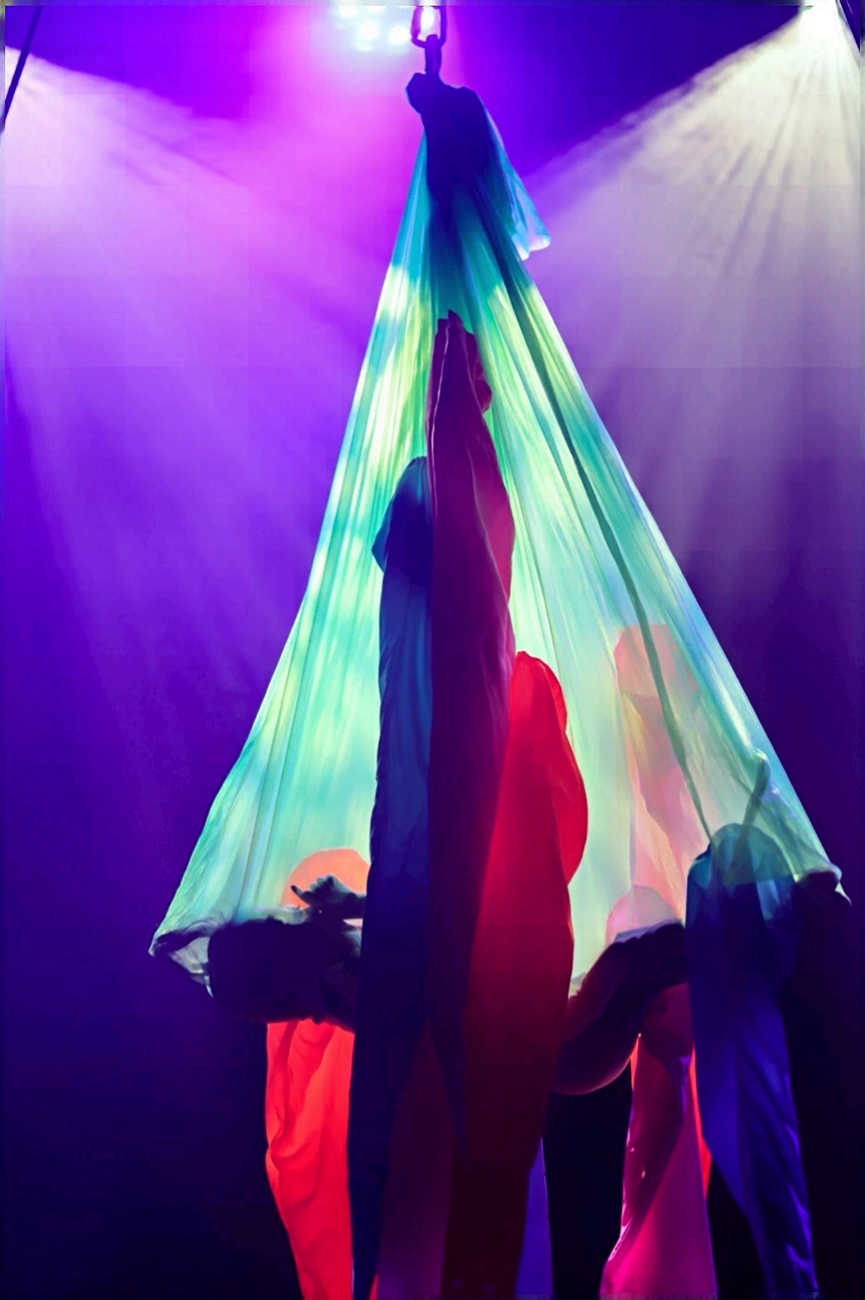
Solo performance by a 6-year-old at “Moonnight Circus 2024” with a guitarist, Showji Kawasaki, who performed at the Opening Ceremony of the Paralympic Games Tokyo.

As a result, he was able to engage in experiences such as “nervousness of a first-time experience,” “ambition to improve,” and “communication,” all of which underscore the significant role that participation plays in his ongoing development and contribute to his psychological growth ^(2)^. Specifically, he showed tangible changes through these experiences: improved physical endurance during travel and performance, growing confidence as he refined his movements after feedback, and enhanced social engagement as he interacted more openly with fellow performers and audiences. His connections with staff and spectators deepened, reflecting an expanding sense of belonging.

The realization of this project required overcoming numerous difficulties. To minimize the risk of aspiration, discussions were repeatedly held among medical staff, the artistic director, the aerial instructor, and the costume designer to determine a posture that was both medically safe and allowed him to move comfortably during aerial sequences. Nevertheless, before and after each performance, the child experienced episodes of increased heart rate, respiratory secretions, and cold-like symptoms such as nasal discharge and cough. Beyond medical considerations, the aerial performance itself involved inherent risks of falling, as the child could not maintain his position in the air through muscular strength. To address this, we worked with welfare equipment specialists to design a cradle-like fabric that fully supported his body. To preserve the acrobatic tension without compromising safety, no belts or rigid harnesses were used. Instead, collaboration with the costume and lighting teams allowed the fabric to remain semi-transparent so that his movements could still be seen through it, balancing both safety and artistic expression. As with our previous experiences ^(3), (4)^, establishing a strong and trusting relationship between the child, their family, and the medical team was a critical foundation for undertaking such a complex challenge.

Moreover, this experience was profoundly meaningful for the child and their family in terms of both psychological and physical development. The family expressed a mixture of anxiety and great pride throughout the process. While they initially worried about the physical strain and public exposure, their concerns gradually turned into joy and confidence as they witnessed the child’s enthusiasm and independence on stage. They shared that being able to participate in all performances with the support of many people gave the child a strong sense of confidence, and although he felt disappointed about missing part of one show due to a pre-event fever, they expressed hope that he would continue to open doors to new and unfamiliar worlds through such experiences. After each performance, they described feeling grateful for the collaboration among medical and artistic teams that made such an experience possible. Indeed, previous studies have demonstrated that opportunities for social participation can empower CMCs and their families, fostering a sense of confidence and helping them to envision future aspirations ^(5)^.

Nonetheless, the existing body of knowledge in this field remains limited. In order to effectively reduce the barriers that prevent CMC and their families from fully engaging with society, it is essential to accumulate more experiential insights through similar, practice-based initiatives. Expanding our understanding of what is achievable―through creative, interdisciplinary collaboration―will be key to shaping a more inclusive society that recognizes and nurtures the potential of every child, regardless of the complexity of their medical needs.

## Article Information

### Acknowledgments

We extend our heartfelt thanks to Akinari Kato for his invaluable support as the photographer for our project. We are also deeply grateful to Keisuke Kanai, the creative director of Moon Night Circus, for his generous support.

### Author Contributions

Conception and design of the study: Emiko Karakawa, Akihiko Ozaki, and Hiroyuki Beniya. Writing this paper, Yudai Kaneda. Critical revision of the paper: Emiko Karakawa, Akihiko Ozaki, Makoto Kosaka, and Hiroyuki Beniya. All the authors read the final draft and approved the submission.

### Conflicts of Interest

Akihiko Ozaki received personal fees from MNES, Kyowa Kirin Inc., Becton, Dickinson and Company, Pfizer, Daiichi Sankyo Inc., and Taiho Pharmaceutical Co., Ltd., outside the scope of the submitted work.

### Consent

Written informed consent for publication of this case report, including the clinical information and accompanying images, was obtained from the patient’s mother.

## References

[1] Trends in policies for supporting children with medical complexity [Internet]. Ministry of Health, Labour and Welfare. 2024 [cited 2025 Ap 15]. Japanese. Available at: https://www.mhlw.go.jp/content/10800000/000584473.pdf

[2] International classification of functioning, disability and health: children and youth version: ICF-CY [Internet]. World Health Organization. 2007 [cited 2025 Ap 15]. Available at: https://iris.who.int/handle/10665/43737

[3] Kosaka M, Murata N, Kaneda Y, et al. Challenges when going on excursions with children with medical complexity in Japan. Pediatr Int. 2023;65(1):e15403. 36318269 10.1111/ped.15403

[4] Miyatake H, Onishi T, Kaneda Y, et al. Possibility for children with medical complexities to reach a 3000-m peak: a report of 2 cases. Wilderness Environ Med. 2023;34(3):383-7. 37438154 10.1016/j.wem.2023.05.008

[5] Kosaka M, Kotera Y, Masunaga H, et al. Emotional impacts of excursions on parents of children with medical complexity. Pediatr Int. 2023;65(1):e15683. 37969062 10.1111/ped.15683

